# Corrigendum: Object-Based Analyses in FIJI/ImageJ to Measure Local RNA Translation Sites in Neurites in Response to Aβ1-42 Oligomers

**DOI:** 10.3389/fnins.2021.792975

**Published:** 2021-11-19

**Authors:** María Gamarra, Maite Blanco-Urrejola, Andreia F. R. Batista, Josune Imaz, Jimena Baleriola

**Affiliations:** ^1^Achucarro Basque Center for Neuroscience, Leioa, Spain; ^2^Department of Neurosciences, Faculty of Medicine and Nursing, University of the Basque Country, Bilbao, Spain; ^3^Department of Cell Biology and Histology, Faculty of Medicine and Nursing, University of the Basque Country, Leioa, Spain; ^4^Life and Health Sciences Research Institute, School of Medicine, University of Minho, Braga, Portugal; ^5^ICVS/3B's, PT Associate Laboratory, Universidade Do Minho, Guimarães, Portugal; ^6^IKERBASQUE Basque Foundation for Science, Bilbao, Spain

**Keywords:** local protein synthesis, RNA localization, neurites, fluorescence microscopy, FIJI/ImageJ analyses, colocalization analyses

In the original article, there was a mistake in Figure 5 as published. In Figure 5F the blue line should correspond to the results reported from DMSO-treated cells and the red line should correspond to the results from Aβ-treated cells. The blue dot from the figure legend should therefore be labelled as “1 DMSO” and the red dot should be labelled as “2 Aβ_1−42_”. The corrected Figure 5 appears below.

**Figure 5:**
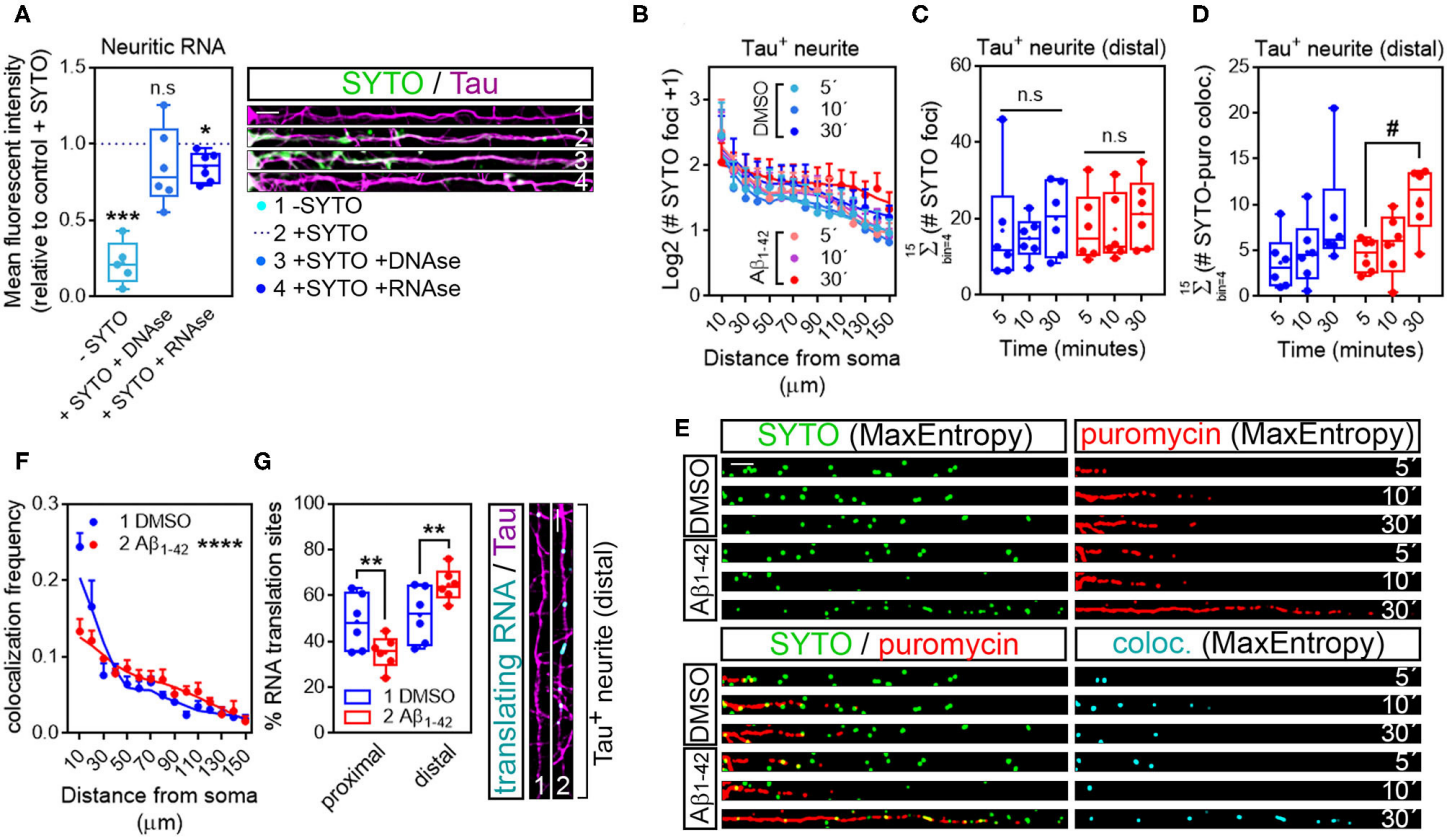
Puromycin-positive foci in axons are a result of local protein synthesis. **(A)** Cells grown for 9 DIV and treated with DMSO for 24 h. Cells immunostained with an anti-Tau antibody (magenta) were incubated with SYTO RNASelect green fluorescent dye to label endogenous RNA (green). Total green fluorescence intensity was measured in neurites covering a distance of 150 μm from the edge of the soma (2, + SYTO). As negative control, green fluorescence was measured in cells that had not been incubated with SYTO (1, -SYTO). To determine if SYTO selectively labeled RNA, some fixed cells were digested with DNAse (3, + SYTO + DNAse) or with RNAse (4, + SYTO + RNAse). Box and whisker graphs represent the average relative fluorescence intensity of 10 neurites per condition, shown as individual data points, and the mean and median of 5 (*n* = 5, − SYTO negative samples compared to their corresponding + SYTO controls) or 6 (*n* = 6, + SYTO + DNAse and + SYTO + RNAse compared to their corresponding + SYTO controls) independent experiments. ***p < 0.001; *p < 0.05; n.s, not significant; two-tailed *t*-tests. Scale bar, 10 μm. **(B)** SYTO-positive staining [as represented in green in **(E)**] from randomly selected cells was filtered with the convolver, brightness and contrast were adjusted. Images were converted to 8-bit and binarized with the MaxEntropy mask. The longest Tau-positive neurite was selected with a segmented line and straighten, smoothen and binarized with the MaxEntropy mask (MaxEntropy). SYTO-positive discrete puncta were scored with the particle analyzer in 15 bins covering a distance of 150 μm from the edge of the soma. The graph shows the average number on puncta represented as Log2 (SYTO foci + 1) vs. distance ± SEM measured in 6 independent experiments (*n* = 6). No statistical differences were detected between DMSO- and Aβ-treated cells incubated with puromycin for 5, 10, or 30 mins. **(C)** Box and whisker graphs show the total number of RNA granules in distal sites of Tau-positive neurites [Σ (# SYTO foci)] from DMSO- and Aβ-treated cells incubated with puromycin for 5, 10, or 30 mins. Data represent the average value of 10 sampled cells per condition plotted as individual data points, and the mean and median of 6 independent experiments (*n* = 6). n.s, no significant; two-way ANOVA followed by Tukey's multiple comparison test. **(D)** Parallel to processing SYTO-labeled images, puromycin staining was filtered with the convolver, brightness and contrast were adjusted. Images were converted to 8-bit and binarized with the MaxEntropy mask. Same Tau-positive neurites selected for SYTO quantification (green channel) were selected in the red channel [puromycin staining in **(E)**], straighten, smoothen and binarized with the MaxEntropy mask. Co-localized objects were obtained with the AND function in the image calculator [cyan in **(E)**] and scored in distal sites of Tau-positive neurites with the particle analyzer. Box and whisker graphs show the total RNA-protein colocalized puncta in DMSO- and Aβ-treated cells incubated with puromycin for 5, 10, or 30 mins [Σ (# SYTO-puro coloc.)]. Data represent the average value of 10 sampled neurites per condition plotted as individual data points, and the mean and median of 6 independent experiments (*n* = 6). #*p* < 0.05 5 vs. 30 min puromycin in Aβ-treated cells; two-way ANOVA followed by Tukey's multiple comparison test. **(E)** Micrographs from straighten, binarized neurites stained with SYTO RNASelect green fluorescent dye to label RNA (green), with an anti-puromycin antibody to label protein (red) and the resulting images when merging both channels (green, red, and yellow) and when combining both with the AND function in the image calculator (cyan). Cells treated with puromycin for 5, 10, or 30 mins are shown. Scale bar, 10 μm. **(F)** The graph represents the frequency distribution of SYTO- and puromycin-positive objects (colocalization frequency) in DMSO- and Aβ-treated neurites following 30-min puromycin exposure. Colocalized objects were measured with the particle analyzer in 15 bins covering a distance of 150 μm from the edge of the cell body. ****p < 0.0001 (interaction); two-way ANOVA. **(G)** Box and whisker graph representing the proportion of colocalized objects (% RNA translation sites) in proximal (0–30 μm) and distal (last 120 μm) sites of Tau-positive neurites. Data represent the average value of 10 sampled cells per condition shown as individual data points, and the mean and median of 6 independent experiments (*n* = 6). **p < 0.01; two-tailed *t*-test. Micrographs show colocalized objects (translating RNAs, cyan) detected within the last 120 μm (distal) of Tau positive neurites (magenta). Scale bar, 10 μm.

The authors apologize for this error and state that this does not change the scientific conclusions of the article in any way. The original article has been updated.

## Publisher's Note

All claims expressed in this article are solely those of the authors and do not necessarily represent those of their affiliated organizations, or those of the publisher, the editors and the reviewers. Any product that may be evaluated in this article, or claim that may be made by its manufacturer, is not guaranteed or endorsed by the publisher.

