# Supplementary figures and images for: Object-Based Analyses in FIJI/ImageJ to Measure Local RNA Translation Sites in Neurites in Response to Aβ1-42 Oligomers
Source: Front Neurosci. 2020 Jun 3;14:547. doi: 10.3389/fnins.2020.00547 (PMC7284234; doi:10.3389/fnins.2020.00547)

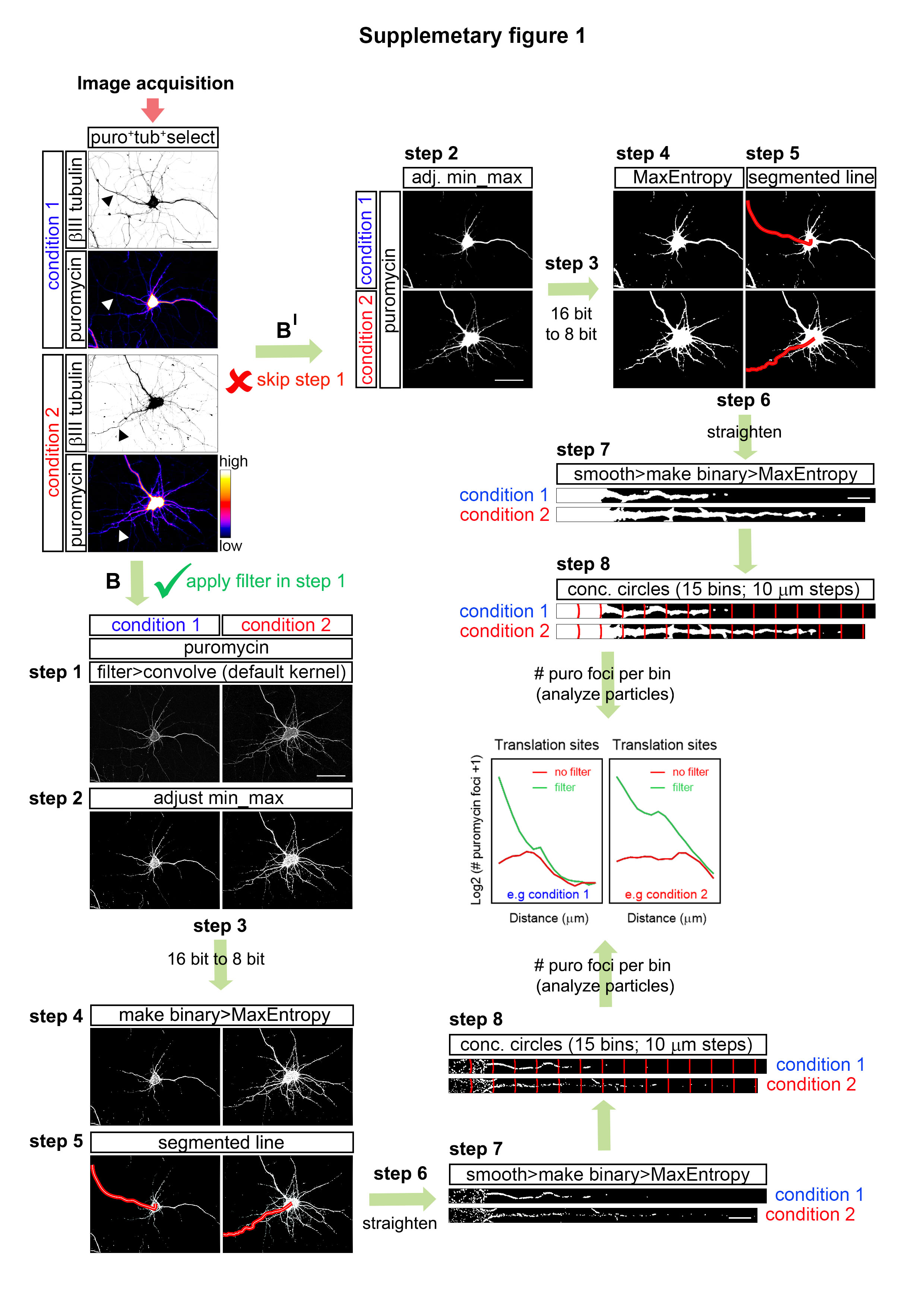

Supplement: FIGURE S1 — Workflows for image processing with or without applying the default convolution kernel (Laplacian filter). Images show the same cells used as examples for the workflow in Figure 1. (B) Represents the same step by step processing method described Figure 1B. (B|) Corresponds to the same workflow as in Figure 1B excluding step 1 which corresponds to the application of the Laplacian filter to enhance the edges. Both workflows converge in graphs comparing both methods (green line, filter application; red line, no filter) in two experimental conditions (condition 1, blue; condition 2, red). Graphs represent Log2(#puromycin foci +1) vs. distance. Scale bars, 50 μm in whole-cell micrographs and 10 μm in straighten neurites. [file Image_1.JPEG]
